# A single hybrid origin of cultivated peanut

**DOI:** 10.1111/tpj.70619

**Published:** 2025-12-24

**Authors:** Francisco J. de Blas, Soraya C. M. Leal‐Bertioli, José G. Seijo, Brian L. Abernathy, Justin Vaughn, Dhanushya Ramachandran, Steven B. Cannon, Josh Clevenger, Brian Scheffler, David J. Bertioli

**Affiliations:** ^1^ Departamento de Fundamentación Biológica, Genética, Facultad de Ciencias Agropecuarias Universidad Nacional de Córdoba Córdoba Córdoba 5001 Argentina; ^2^ Institute of Plant Breeding, Genetics and Genomics University of Georgia Athens 30602 Georgia USA; ^3^ Department of Plant Pathology University of Georgia Athens 30602 Georgia USA; ^4^ Instituto de Botánica del Nordeste (IBONE‐UNNE‐CONICET) Consejo Nacional de Investigaciones Científicas y Técnicas and Universidad Nacional del Nordeste and Consejo Nacional de Investigaciones Científicas y Técnicas Corrientes Corrientes 3400 Argentina; ^5^ Facultad de Ciencias Exactas y Naturales y Agrimensura, Universidad Nacional del Nordeste Corrientes 3400 Argentina; ^6^ Genomics and Bioinformatics Research Unit, US Department of Agriculture—Agricultural Research Service Stoneville Mississippi USA; ^7^ Corn Insects and Crop Genetics Research Unit, US Department of Agriculture—Agricultural Research Service Ames 50011 Iowa USA; ^8^ HudsonAlpha Institute for Biotechnology, Alabama Huntsville Alabama 35806 USA; ^9^ Department of Crop & Soil Sciences University of Georgia Athens 30602 Georgia USA

**Keywords:** peanut, groundnut, *Arachis hypogaea*, *Arachis duranensis*, *Arachis ipaënsis*, *Arachis monticola*, polyploid origin, domestication, crop evolution

## Abstract

This study, the first in a three‐part series, lays the foundation for understanding the origin of the peanut crop (*Arachis hypogaea*). Its subsequent evolution is explored in the two papers that follow. The evidence that *A. hypogaea* originated from a single hybridization event between *Arachis duranensis* and *Arachis ipaënsis* less than 10 000 years ago was already very strong. Here, we extend this evidence using more than 1600 single‐nucleotide polymorphisms to make an almost exhaustive comparison of wild *Arachis* section germplasm conserved *ex situ* with the A and B subgenomes of divergent, sequenced cultivated peanuts. The wild relatives of peanut are highly selfing and their geocarpy means they plant their own seeds, allowing them to persist as discrete populations for millennia. This unusual biology creates a rare opportunity for genetic archaeology: ancestral lineages can be identified with exceptional precision. Our results reaffirm a single origin for the cultigen, identifying *A. duranensis* from Río Seco and *A. ipaënsis* K 30076 as the closest known relatives of the A and B subgenomes of peanut. As a genomic resource, we generated a chromosome‐scale assembly of the Río Seco *A. duranensis* K 30065 and confirmed that it is more closely related to the A subgenome of peanut than the current reference genome (V14167). Even if somewhat closer wild accessions were found through new field collections, they would still belong to the same ancestral lineage. With this level of evidence, the origin of peanut is now known in greater detail than that of any other ancient polyploid crop.

## Significance Statement

We identify with exceptional precision the wild ancestors of cultivated peanut and reaffirm its origin from a single hybridization event, based on high‐resolution DNA comparison with nearly all *ex situ* accessions of wild species closely related to peanut. This work makes peanut the best‐understood ancient polyploid crop in terms of its evolutionary origin.

## INTRODUCTION

The genus *Arachis*, native to South America, is distributed across diverse environments. It currently comprises 84 described species, which are classified into nine sections based on morphological, geographical, and cross‐compatibility characteristics (Krapovickas & Gregory, 1994; Santana & Valls, 2015; Seijo et al., 2021, 2025; Valls & Simpson, 2005, 2017). The use of *Arachis* species for food has long been documented, by far the most economically important being the domesticated peanut, *Arachis hypogaea* L., a staple crop throughout the tropics and subtropics (FAOSTAT, 2023). It has two subspecies *hypogaea* and *fastigiata*, with prostrate and upright growth habits respectively, six botanical varieties and thousands of landraces and cultivars with diverse seed colors and pod shapes (Krapovickas et al., 2009, 2013, 2021; Krapovickas & Gregory, 1994). But where, and how, did it originate?

Archaeological evidence from coastal Peru, where the dry climate favors the preservation of plant remains, indicates a long history of peanut use and cultivation in that region. Macrofossils and starch grains dated to as early as 10 000 years ago can be attributed to the cultivation of wild *Arachis* species (Dillehay et al., 2007; Piperno, 2011). The earliest remains positively identified as the domesticated species *A. hypogaea* were found at Middle Preceramic sites dating to around 5000 years ago in Quebrada Tierra Blanca, Peru; the distinctive reticulated patterns on the pod shells identify them as belonging to the *hirsuta* botanical variety (Rossen et al., 1996). Similar reticulated pods have been recovered from other dry climate archaeological sites in Peru, Chile, and Argentina. These same *hirsuta* pods are also represented in the dramatic gold and silver high‐status necklace from the Moche royal tombs of Sipán, dating to about 2500 years ago, highlighting peanut's ritual and social significance (Masur et al., 2018). Landraces with these ancestral traits are still found in local Peruvian markets today. While *hirsuta* is the best documented in the archaeological record, other botanical varieties of *A. hypogaea* were likely cultivated from similarly early dates in regions east of the Andes, where wetter conditions are much less favorable for the preservation of archaeological plant material.

**Figure 1:**
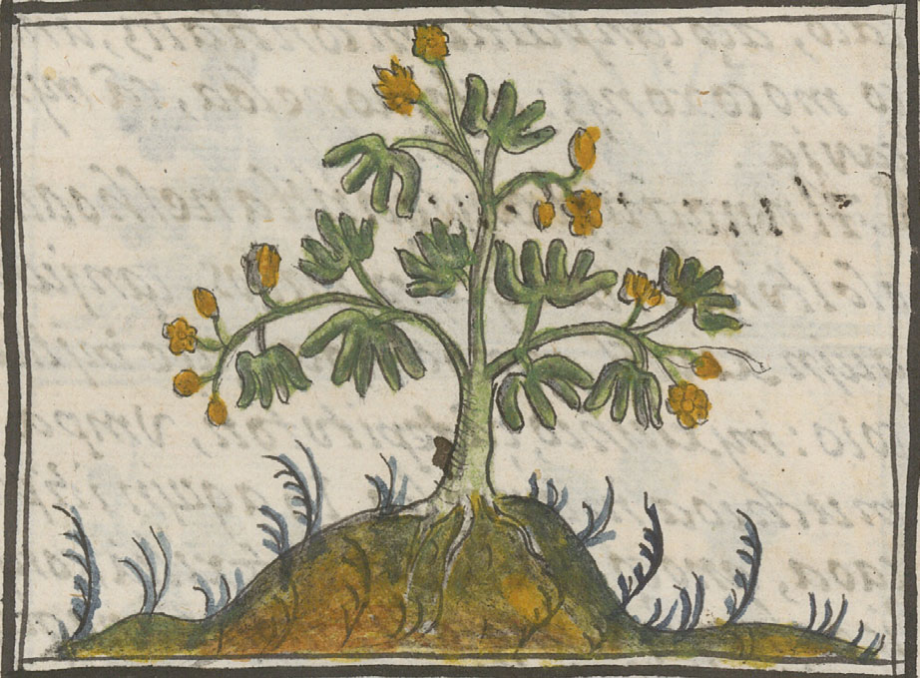
Illustration of peanut plant (*tlalcacahuatl*) drawn in 1569, General History of the Things of New Spain by Fray Bernardino de Sahagún: The Florentine Codex. Book XI: Natural Things, Folio 140v. Medicea Laurenziana Library, Florence, World Digital Library.

Following the peanut's initial domestication and dispersal in South America, its pre‐Columbian history in Mexico was long unclear. The scarcity of mentions in major historical codices and conquest reports, such as the detailed work of Friar Bernardino de Sahagún who noted its Nahuatl name (tlalcacahuatl) only for medicinal use and not as a food source, suggested it was of little importance in central Mexico (Hammons et al., 2016). This led to the assumption that it was a post‐conquest introduction by the Spaniards. However, this view was overturned by archeobotanical evidence. The recovery of domesticated peanut remains from the Coxcatlán dry caves in the Tehuacán Valley, dating to 2200 years ago, confirms its presence as a cultigen in the region centuries before European contact (MacNeish, 1965; Smith, 1967). Critically, however, its limited abundance in archeological contexts aligns with its scant historical record, indicating that while the peanut was present in pre‐Columbian Mexico, it remained a minor crop of localized significance, never achieving the status of a staple in core Mesoamerican agriculture like maize or beans. Historical chronicles of the Americas provide the earliest written accounts of the Andean origins of peanuts, such as the record of Inca Garcilaso de la Vega, who in 1609 documented the pre‐Columbian cultivation of peanuts, known to the Inca indigenous people as *ynchic*. This marked the beginning of a series of observations by other chroniclers underscoring the broad distribution and importance of peanut from the Atlantic to the Pacific coast of central and southern South America. The Florentine Codex, produced around 1577 by the ethnologist Fray Bernardino de Sahagún (Sahagún, 1577) contains a drawing, made in 1569, in which a peanut plant, known as *tlalcacahuatl* in the Aztec language, is depicted (Figure 1).

**Figure 2:**
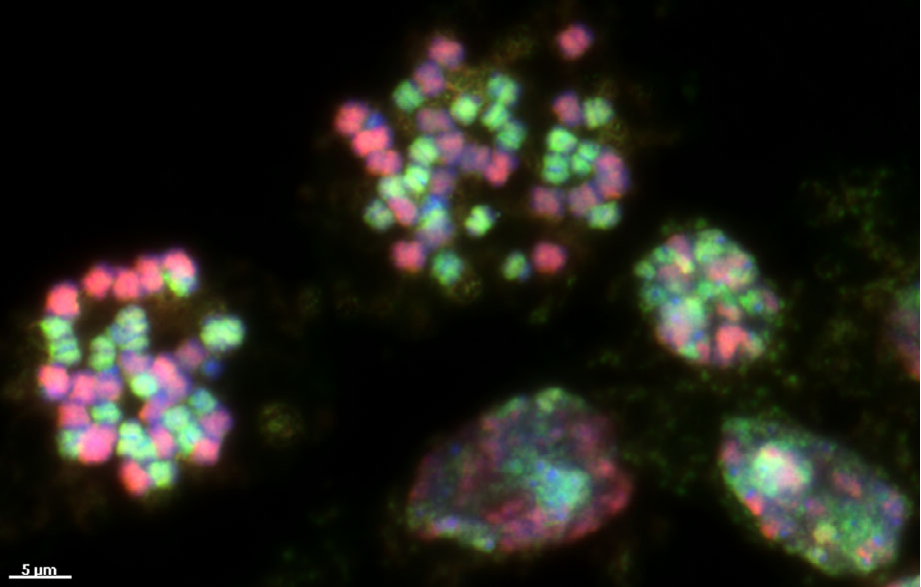
Genomic *in situ* hybridization image illustrating the hybrid nature of cultivated peanut (*Arachis hypogaea*), showing the presence of two distinct parental genomes within a single nucleus. The image, courtesy of Aiko Iwata‐Otsubo, highlights chromosomal contributions from *Arachis duranensis* (A genome, green) and *Arachis ipaënsis* (B genome, red). [As is typical of chromosome squashes, where breakage and overlap are common, the expected number of chromosomes is not clearly visible. It is standard practice to select images that most closely reflect the most typical karyotype. While this is a reasonable convention, it can also obscure the occurrence of chromosomal abnormalities.]

While nearly all wild *Arachis* species are diploid, the domesticated peanut, *A. hypogaea*, is a tetraploid, and like most crops, is not found growing in the wild. Understanding how this tetraploid species arose has been of great interest in peanut research. Decades of cytogenetic, molecular, and biogeographic studies have converged on a consistent conclusion that peanuts originated east of the Andes, encompassing southeastern Bolivia and northwestern Argentina from hybridization between two wild diploid species, *Arachis duranensis* and *Arachis ipaënsis* (Fernández & Krapovickas, 1994; Krapovickas, 1995; Kochert et al., 1996; Simpson et al., 2001; Fávero et al., 2006; Seijo et al., 2007; Robledo et al., 2009; Robledo & Seijo, 2010; Grabiele et al., 2012; Moretzsohn et al., 2013; Leal‐Bertioli et al., 2015; Bertioli et al., 2016; Bertioli et al., 2019). This hybrid origin, resulting in the presence of two distinct genomes within a single nucleus, is illustrated in Figure 2.

The peculiar biogeography of the diploid progenitors points to human‐mediated dispersal. While *A. duranensis* thrives across a considerable geographic range in the eastern foothills of the Andes and dry Chaco in Argentina, Bolivia, and Paraguay, *A. ipaënsis* has only ever been found in a single location near Villa Montes in Bolivia. This site is within the range of *A. duranensis* but is geographically isolated from the range of *A. ipaënsis*'s most closely related species, strongly suggesting it was transported there in prehistory (Krapovickas et al., 2009; Krapovickas & Gregory, 1994). Human involvement in the origin of the tetraploid is further supported by the biogeography of *Arachis monticola*, the wild form of cultivated peanut. It is found only in two small areas in the eastern foothills of the Andes, in northwest Argentina, the Río Grande de Jujuy, and Lerma valleys, both associated with early human settlements. Moreover, *A. monticola* occurs at higher elevations than any diploid *Arachis* species, making natural dispersal to these sites extremely unlikely (Krapovickas & Gregory, 1994). Recent genomic evidence has clarified that *A. monticola* is not a feral form but the wild representative of the allotetraploid lineage sharing an origin with *A. hypogaea* (Bertioli et al., 2019). Thus, the distributions of both key diploids and the wild tetraploid, corroborated by genome sequences, converge on a scenario of a single hybrid origin facilitated by human movement of species.

Archaeological finds support that wild *Arachis* species with pod characteristics consistent with *A. duranensis* (smooth shells) and *A. ipaënsis* (reticulated shells) were cultivated in close proximity (Simpson et al., 2001). These findings come from coastal Peru, where dry conditions favor the preservation of plant materials. However, the cultivation and movement of wild *Arachis* east of the Andes is well supported by the current presence of ruderal populations of wild *Arachis* growing near ancient human settlements (see supplementary note 1 of Bertioli et al., 2019). In this region, the ancestral species would have been brought into close proximity by early human activity and ecological conditions were favorable for serendipitous cross‐pollination by a native bee, setting in motion the evolutionary adventure of peanut (Simpson et al., 2001).

The identity of the ancestral diploids is strongly supported by complementary lines of evidence. Fernández and Krapovickas (1994) used classical cytogenetics and Kochert et al. (1996) used molecular markers to trace the genetic origins of *A. hypogaea* to *A. duranensis* and *A. ipaënsis*. Crossability studies provided compelling experimental support for the very close affinities between *A. duranensis*, *A. ipaënsis*, and cultivated peanut: a tetraploid hybrid (referred to as an amphidiploid in the original study) formed from the diploids was successfully crossed with all six botanical varieties of *A. hypogaea*, producing fertile hybrids (Fávero et al., 2006; Simpson, 2024). Bertioli et al. (2016) reported that a comparison of segregation distortion in mapping populations from different studies further reinforces this close relationship. Populations derived from crosses between cultivated peanut and the tetraploid hybrid show lower levels of genetic distortion than those from some crosses within *A. hypogaea*. Furthermore, the chromosomes of *A. hypogaea* show the highest DNA complementarity with *A. duranensis* and *A. ipaënsis*. This was shown using double genomic *in situ* hybridization (GISH), in which differentially labeled DNA from candidate diploid species is hybridized to polyploid chromosomes to assess the affinity of complementary DNA sequences (Seijo et al., 2007). Comparative analyses of chromosome morphology and structure showed that from a selection of A and B subgenome species, *A. duranensis* and *A. ipaënsis* very closely resemble the respective chromosomes of cultivated peanut (Robledo et al., 2009; Robledo & Seijo, 2010). Genetic analyses by Moretzsohn et al. (2013) that separated intron sequences from the A and B subgenomes of cultivated peanut showed that *A. ipaënsis* is strikingly similar to the B subgenome, while *A. duranensis* forms a group with the A subgenome. Using chloroplast DNA and 5S rDNA, Grabiele et al. (2012) identified a population of *A. duranensis* from Río Seco (Se 2741), Salta, Argentina, as the closest match to the A genome lineage of *A. hypogaea*, and that it may have acted as the female parent. Whole‐genome sequencing further confirmed these findings: *A. ipaënsis* is nearly identical to the B subgenome of cultivated peanut, and a wide survey of *A. duranensis* accessions confirmed those from Río Seco as the closest match to the A subgenome. As a final layer of evidence, ‘fingerprint’ recombination patterns at the ends of chromosomes between the A and B subgenomes are shared across divergent cultivated peanuts and their wild counterpart *A. monticola*—fossilized genomic traces pointing to their common single origin (Bertioli et al., 2016, 2019); and notably different where modern introgression from *Arachis cardenasii* has occurred via crop improvement (Bertioli et al., 2021).

This substantial body of evidence still offered an opportunity for further advance. Wild peanuts are highly selfing and plant their own seeds, allowing them to persist in the same sites for millennia. This unusual biology creates a rare opportunity for genetic archeology: ancestral lineages remain traceable with exceptional precision. Building on this foundation, we conducted a near‐exhaustive analysis—the first of a three‐part series—comparing more than 1600 single‐nucleotide polymorphisms (SNPs) from dissected A and B subgenomes of sequenced, taxonomically divergent cultivated peanut genotypes with array data from nearly all *ex situ* conserved accessions of wild species in the *Arachis* section. This approach allowed us to identify, with exceptional precision, the closest known relatives of both subgenomes, reaffirming a single hybridization event involving *A. duranensis* with *A. ipaënsis*. With this level of genomic resolution and sampling completeness, peanut's origin is now known with rare certainty—more clearly than that of any other ancient polyploid crop.

## RESULTS

To clarify the genetic origins of cultivated peanut with high resolution, we integrated SNP datasets from the dissected A and B subgenomes of five *A. hypogaea* genome assemblies with Axiom_Arachis v02 array data from 272 curated wild accessions—representing nearly all accessions conserved *ex situ* from species of section *Arachis*. This near‐exhaustive comparison identified the closest known relatives of both subgenomes. To add further resolution and provide a new genomic resource, we also produced a chromosome‐scale genome assembly of *A. duranensis* K 30065 from Río Seco and compared it with the reference A genome V 14167 (Bertioli et al., 2016) and the A subgenome of *A. hypogaea*. Genome‐wide variant mapping, principal component analysis (PCA), and phylogenetic analyses supported *A. duranensis* from Salta Province, particularly Río Seco, and *A. ipaënsis* as the closest known relatives of the A and B subgenome of *A. hypogaea*, respectively.

### Concordance analysis between axiom and *in silico* SNPs

To assess the concordance of SNP calls derived from two very different methods—dissected genome assemblies and the Axiom_Arachis v02 genotyping array—we compared *in silico* SNP calls from five wild species genome sequences with corresponding array data generated from DNA extracted from the same accessions (although necessarily different plants). Concordance was greater than or equal to 88.4% in all comparisons. The lowest match was for *Arachis stenosperma*, which shared 88.4% of SNP calls between the *in silico* data (step.V10309_A) and the Axiom array data (step_V10309_PI666100_A; Leal‐Bertioli et al., 2024).

### PCA and phylogenetic analyses

**Figure 3:**
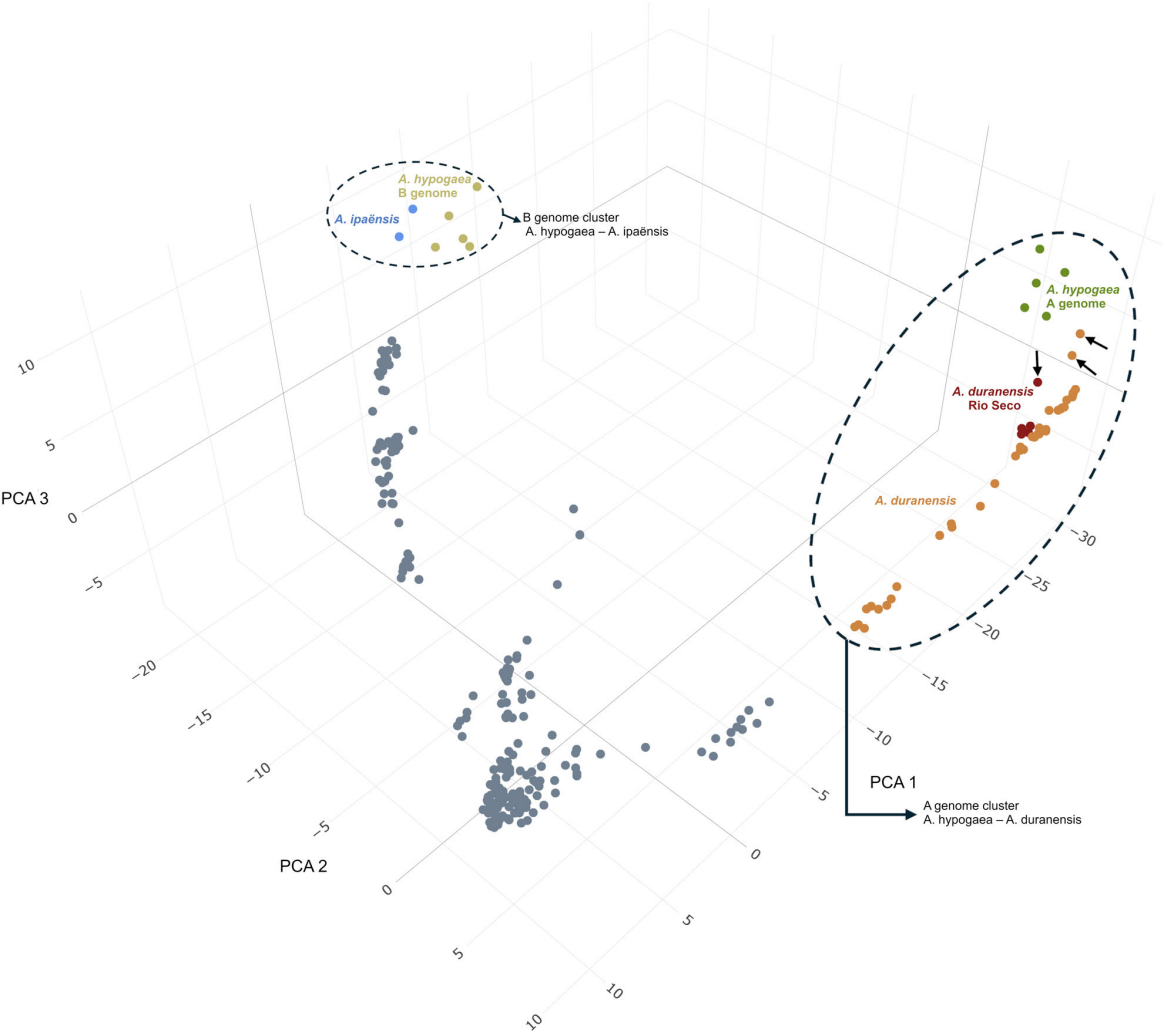
Principal component analysis of 286 samples, including 276 accessions of wild diploid *Arachis* species and five cultivated A and B genome SNP sets each. Samples are colored according to species groups as indicated by labels in the plot. The first three principal components (PC1: 53.49%, PC2: 35.79%, and PC3: 6.46% of variance) reveal that the dissected A and B genomes cluster with *Arachis duranensis* and *Arachis ipaënsis*, respectively. Within the A genome cluster (indicated by a dashed ellipse), the accessions K 30065, V 14167, and K 30060—indicated by black arrows—appear closest to the A genome of *Arachis hypogaea* accessions.

PCA based on the Euclidean distances was performed on 286 accessions, including the dissected A and B subgenomes from *A. hypogaea* (Figure 3).

The projection on PCA 1 (53.49%), PCA 2 (35.79%) and PCA 3 (6.46%) showed that the dissected A and B subgenomes clustered with *A. duranensis* and *A. ipaënsis*, respectively. The projection depicted that cultivated tetraploids dissected A subgenome are closest to the *A. duranensis* accessions, positioning closely to *A. duranensis* K 30065, and the cultivated tetraploids dissected B subgenome are closest to the *A. ipaënsis* K 30076 accession.

**Figure 4:**
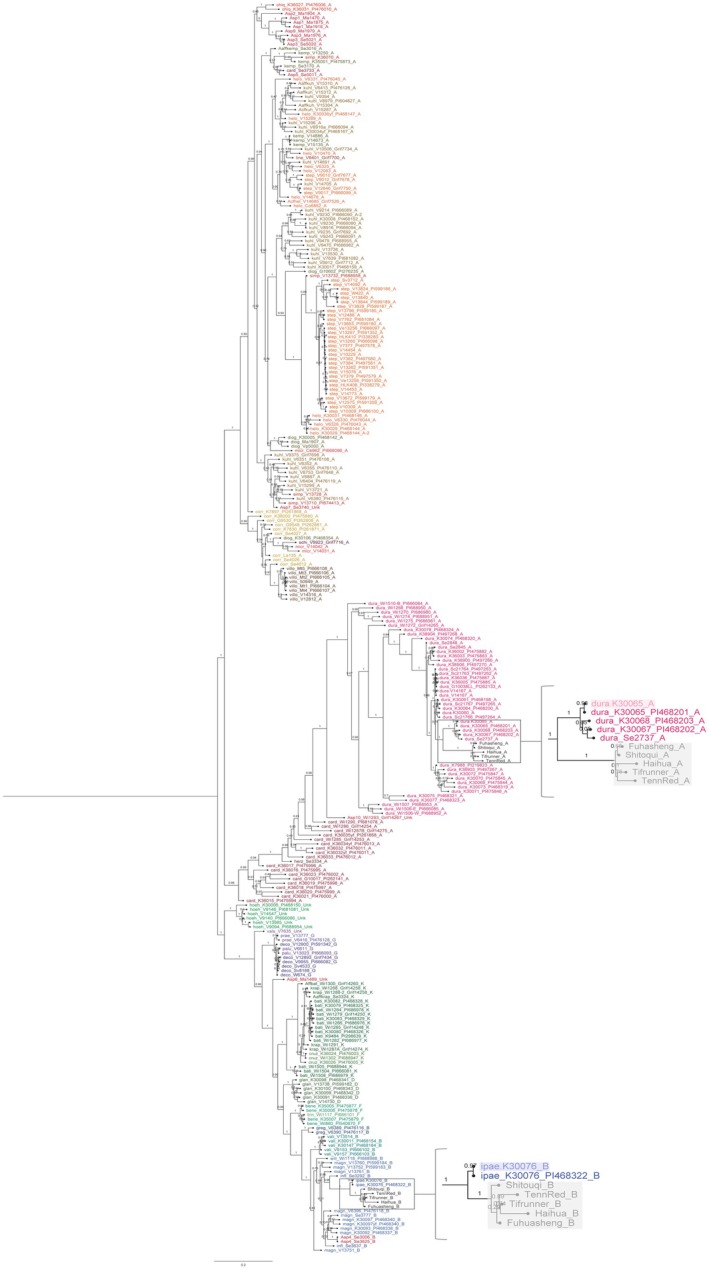
Phylogenetic tree of the 286 samples assessed in this study, highlighting the two clades where *Arachis hypogaea* clusters. The *A. hypogaea* A genome clusters with *Arachis duranensis*, particularly with accessions from Salta, Argentina (Río Seco region), previously reported as the closest to the *A. hypogaea* A genome (Grabiele et al., 2012; Bertioli et al., 2019). For the B genome grouping, *Arachis ipaënsis* K30076 is the accession with which the *A. hypogaea* B genome clusters, consistent with previous reports (Bertioli et al., 2016). For visualization purposes, both sections of the tree are zoomed in, and the *in silico* extracted samples are highlighted with shaded colors.

To investigate the genetic relationship between samples, phylogenetic trees were constructed using the maximum likelihood model. The trees showed consistent clustering of the cultivated A subgenome closest to the Río Seco accessions of *A. duranensis,* including K 30065. The cultivated B subgenome sequences clustered with *A. ipaënsis* K 30076. This clustering was assigned the strongest possible support (bootstrap value = 1) in both cases (Figure 4).

### The genome of *A. duranensis* K 30065

The genome assembly of *A. duranensis* K 30065 is high quality and well resolved, with a total size of 1099 Mb. Of this, 1086 Mb is anchored in 10 pseudomolecules, with 64 within‐chromosome gaps and 112 unanchored scaffolds totaling 13 Mb. Compared with V 14167, the K 30065 assembly shows greater collinearity with the A subgenome of cultivated peanut, offering a slightly more accurate representation of its ancestral lineage and providing a valuable genomic resource for peanut research.

### Variant analysis

**Figure 5:**
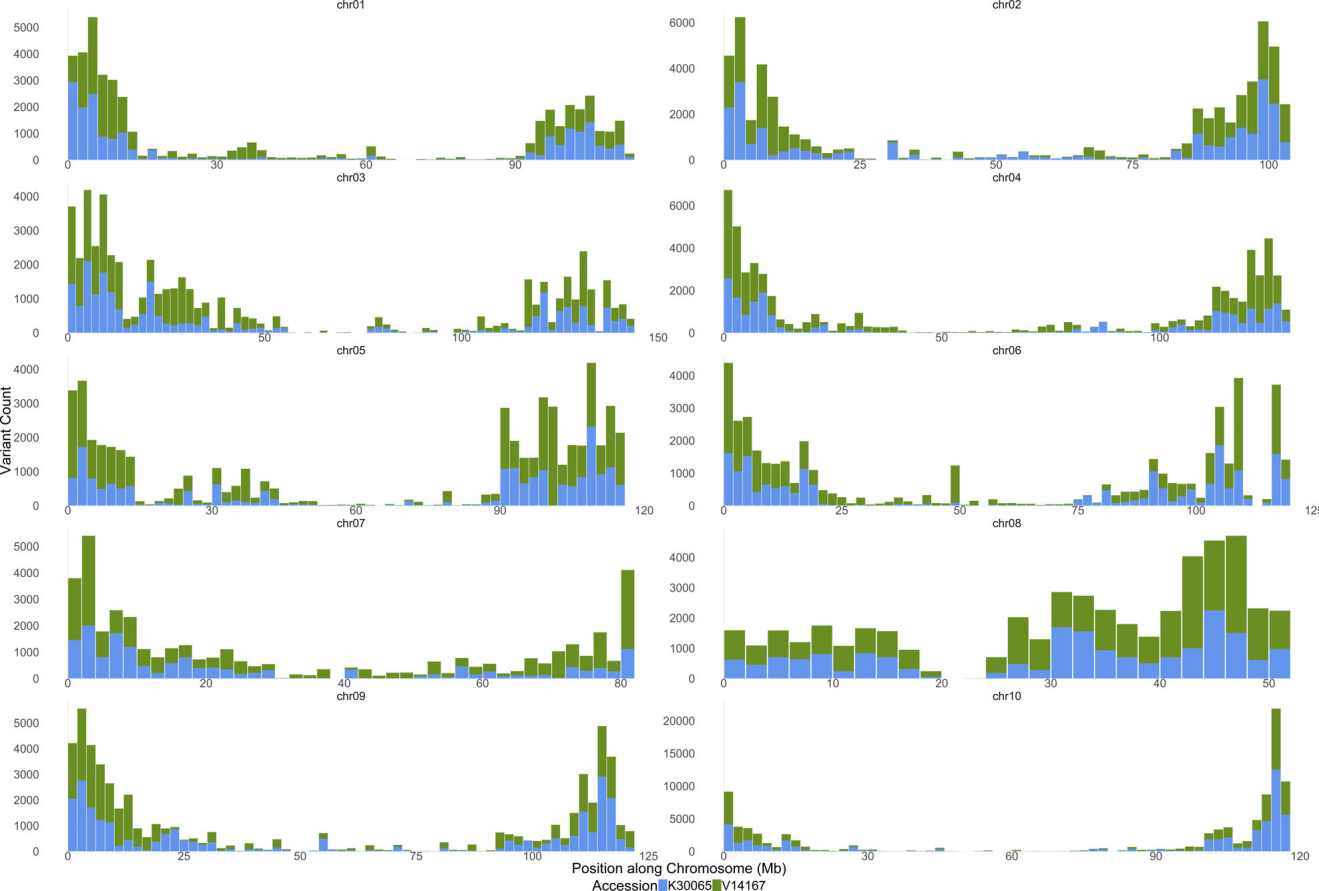
Lineage‐specific variant density per 2 Mb window in *Arachis duranensis* V14167 (green) and K30065 (blue) plotted along the Tifrunner A genic space (represented in stacked‐bar plots), where the *x*‐axis represents position in each chromosome and the *y*‐axis represents variant count.

The genome of *A. duranensis* K 30065, sequenced as part of the USDA's Peanut Genome Initiative, is being used here for the first time in a genome‐wide analysis. We performed whole‐genome multiple sequence alignments using K 30065, V 14167, and the peanut reference genome Tifrunner (subgenome A). These alignments enabled the identification of genomic variants, including SNPs and indels, with the largest variant spanning 167 146 bp. Specifically, we identified 1.81 million lineage‐specific variants for K 30065 and 3.01 million for V 14167. When these variant counts were plotted in 2 MB windows across the genome, K 30065 appeared as the closest ancestor to Tifrunner, with fewer variations compared with V 14167. However, it is important to note that the V 14167 genome was assembled using different sequencing methods (Illumina with Hi‐C) compared with K 30065 (CCR PacBio) (Figure S1). To reduce potential bias, variant counts were also compared exclusively within Tifrunner genic regions, where both technologies are expected to be highly accurate. After this filtering, K 30065 and V 14167 exhibited 238 731 and 315 869 lineage‐specific variants, respectively (Figure 5). Despite some local exceptions, K 30065 consistently showed greater similarity to Tifrunner across a larger number of windows and global divergence measures. Alignment of the three genomes at orthologous positions confirmed *A. duranensis* K 30065 as closer to the A subgenome of cultivated peanut than *A. duranensis* V 14167.

## DISCUSSION

Building on decades of research and integrating genome‐scale datasets from taxonomically divergent accessions, our study reaffirms that *A. hypogaea* arose from a hybridization between *A. duranensis* and *A. ipaënsis*. By analyzing SNP variants from the dissected A and B subgenomes of divergent *A. hypogaea* accessions and comparing them to nearly all known wild *Arachis* section accessions, our analysis achieves near‐exhaustive scope.

Efforts to resolve peanut's origin have been complicated by its tetraploid genome, comprising distinct A and B subgenomes. Kochert et al. (1996) addressed this by inferring the subgenomic origin of RFLP bands *post hoc*—a thoughtful and innovative approach. Despite this early and clear framing of the problem, many subsequent studies treated the tetraploid as a single genetic entity—a fundamentally unsound approach, since an allotetraploid cannot be accurately represented by a single position on a non‐reticulated phylogeny. Moretzsohn et al. (2013) returned to the correct allotetraploid framework, extending it using a broader range of species and more data‐rich intron sequences. The advent of genome sequencing allowed this principle to be applied with vastly more data. Whole‐genome sequences revealed extraordinarily high identity between the B subgenome of *A. hypogaea* and *A. ipaënsis* (Bertioli et al., 2016). Molecular dating estimated divergence at less than 10 000 years, although post‐polyploidy genetic flux between subgenomes—that is, homoeologous recombination between the A and B subgenomes (Bertioli et al., 2016, 2019, 2021)—likely inflates this figure. A similar genome‐scale approach applied to the A subgenome indicated that its closest relatives are *A. duranensis* accessions from Río Seco, Argentina (Bertioli et al., 2019). In the present study, we integrate genome‐scale SNP data from dissected subgenomes of taxonomically divergent sequenced *A*. *hypogaea* accessions with curated array data from nearly all known wild accessions of section *Arachis* (Leal‐Bertioli et al., 2024)—merging data types to produce the most comprehensive assessment of peanut's origin to date. The A subgenomes of *A. hypogaea* form a sister group to the Río Seco *A. duranensis* accessions, and the B subgenomes to *A. ipaënsis*, each with maximal support (bootstrap = 1). Notably, SNP markers from genomic DNA revealed that the *A. duranensis* accession K30060 is closely related to Río Seco accessions, despite being collected 180 km to the south in Río Perico, Jujuy province in Argentina. This finding reinforces a genetic link between the Río Seco population and other regions where previous studies have reported high chloroplast DNA similarity (Grabiele et al., 2012) and further supports the human‐mediated movement of *A. duranensis* during the events that gave rise to cultivated peanut.

The close relationships between the wild diploids and the subgenomes of *A. hypogaea* persist even though genetic flux between subgenomes must, to some extent, erode phylogenetic signal at individual loci. However, genome‐scale datasets such as ours retain ample phylogenomic signal to consistently recover *A. duranensis* and *A. ipaënsis* as the closest relatives of the A and B subgenomes, respectively. We strengthened these conclusions by generating a high‐quality, chromosome‐scale genome assembly of an *A. duranensis* accession from Río Seco (K 30065), which shows higher similarity to the A subgenome of *A. hypogaea* than the current A genome reference.

Although our sampling of wild *Arachis* accessions is near‐exhaustive, the sampling of *A. hypogaea* genotypes is necessarily limited to the available sequenced genomes. Nevertheless, multiple lines of evidence indicate that these genotypes adequately capture the cultivated diversity relevant to assessing peanut's origin. Taxonomically, they represent both subspecies—*hypogaea* and *fastigiata*—and *A. hypogaea* is well known to have remarkably low DNA polymorphism (e.g., Kochert et al., 1996; Korani et al., 2019). Phylogenetic analysis of 791 cultivated peanut accessions, carefully chosen by Otyama et al. (2020) to represent the variation within the species, further supports this point. Although two groups of accessions with some notable primitive characteristics are placed more basally than our sampled genotypes, they cluster even closer to the reconstructed prototype peanut (*in silico* hybrid of *A. ipaënsis* × *A. duranensis*), reinforcing further a single origin.

Additional independent evidence comes from patterns of homoeologous recombination and has been previously documented. At both the scale of large distal chromosome segments and fine‐scale recombination fingerprints at chromosome termini, cultivated peanuts (and *A. monticola*) consistently share homologous exchange signatures (Bertioli et al., 2016, 2019, 2021). These ‘fossilized’ recombination patterns—shared breakpoints of component subgenome recombinations across divergent *A. hypogaea* and *A. monticola* accessions—provide strong evidence of a single hybridization origin. Independent events would not converge on identical recombination signatures across diverse lineages.

Our near‐exhaustive sampling of wild *Arachis* accessions and the well‐documented narrow genetic diversity of cultivated peanut underpin a conclusion of exceptional strength. Nevertheless, all retrospective analyses have formal logical limitations—it is impossible to prove a negative. Although spontaneous introgression (i.e., diploid to tetraploid genetic flow without human mediation) is theoretically possible (Bartolić et al., 2024), none has been detected in peanut to date, and even if such events had occurred, they would not alter the primary ancestry of *A. hypogaea*. Similarly, while we cannot exclude the possibility that *A. hypogaea* originated from a number of closely related tetraploids that originated from the same parental A and B populations that later underwent a genetic bottleneck of a single plant, this would similarly leave the primary ancestry unchanged. Any extant or extinct hypothetical closer relative of the A or B genome would necessarily belong to the same evolutionary clades already identified—*A. duranensis* from Río Seco for the A subgenome and *A. ipaënsis* for the B subgenome—leaving the fundamental inference intact.

Despite this exceptionally narrow and recent origin, cultivated peanut diversified into two subspecies, six botanical varieties, and thousands of landraces, with distinct and varied growth habits, pod shapes, and seed colors and sizes. This paradox—how such rich phenotypic diversity arose from such limited genetic input—is the focus of the second paper in this series (Lamon et al., 2025a). There, we use a synthetic neoallotetraploid to demonstrate how polyploidy itself enabled phenotypic diversification and enhanced responsiveness to selection. In the third and final study of this series, we examine whether the polyploid‐related instability demonstrated in the neotetraploids is still found in modern cultivated peanut (Lamon et al., 2025b).

## MATERIAL AND METHODS

This research builds on previous work done in the Wild Peanut Lab at the University of Georgia, which developed a tree of genetic relationships representing almost all the accessions of wild species conserved *ex situ* belonging to botanical section *Arachis* with unprecedented scope and detail (Leal‐Bertioli et al., 2024).

### Analysis of *in silico* SNP detection quality

We utilized a set of 276 well‐identified and non‐redundant wild *Arachis* species accessions from the *Arachis* section, as characterized in Leal‐Bertioli et al. (2024) (Table 1). These accessions were genotyped using a curated database of 13 000 SNPs derived from the 47K SNP Axiom_Arachis v02 genotyping platform (Korani et al., 2019). This study presents a comprehensive examination of all *Arachis* collections made since 1941 and preserved *ex situ*, resulting in a genetic structure analysis and a phylogenetic tree that includes a curated set of the most up‐to‐date conserved accessions. The curated SNP dataset served as a reference panel, against which SNP data from five *A. hypogaea* accessions—encompassing the botanical varieties *hypogaea*, *fastigiata*, and *vulgaris*—were aligned (Table 2). High‐quality genome sequences for each of these cultivated accessions were employed to perform *in silico* SNP detection as follows. Candidate 71 bp (1 pair of SNP alleles and 35 bp flanking sequences) 47K SNP Axiom_Arachis v02 (Korani et al., 2019) microarray probe sets were collected and filtered to remove duplicate and non‐full length probes. The remaining target probe sequences (for both probe alleles e.g., Axiom probe seq: …CAGTCAGT[A/C]CAGTCAGT…; probe allele1 seq: …CAGTCAGTACAGTCAGT…; probe allele2 seq: …CAGTCAGTCCAGTCAGT…) were then aligned to an *Arachis* genome using minimap2 v2.24‐r1122. The alignments were filtered to remove non‐full length alignments as well as alignments that included indels, allele mismatches, or more than 1 flanking sequence mismatch per site. If a single probe alignment remained after filtering, the associated allele info (allele base, position, strand orientation and left/right mismatch count) was recorded for further analysis. This process was repeated for each *Arachis* genome included in the study. The *in silico* SNPs identified in the five *A. hypogaea* accessions were then aligned to the diploid genomes. For the tetraploid genomes, the A and B subgenomes were processed separately, providing distinct SNP data for each genome component of the tetraploid species.

**Table 1:** Set of 276 well‐identified and non‐redundant wild *Arachis* species accessions from the *Arachis* section, as characterized in Leal‐Bertioli et al. (2024). Information includes Sample name as shown in this work, Germplasm bank in which the sample is deposited, Species name, Genome, and Growth habit

		Classification		
Sample name (Figures 5 and 6)	Source	Species	Genome	Lifespan
dura.K30060_A	GRIN‐USDA	*A. duranensis*	A	Annual
dura.K30065_A	GRIN‐USDA	*A. duranensis*	A	Annual
dura.V14167_A	GRIN‐USDA	*A. duranensis*	A	Annual
helo_Co6862_A	Embrapa	*A. helodes*	A	Perennial
Aaffkemp_Se3016_A	IBONE	*A*. aff. *kempff‐mercadoi*	A	Perennial
Aaffkrap_Se3324_K	IBONE	*A*. aff. *krapovickasii*	K	Annual
Aaffkuh_V15304_A	Embrapa	*A*. aff. *kuhlmannii*	A	Perennial
Aaffkuh_V15310_A	Embrapa	*A*. aff. *kuhlmannii*	A	Perennial
Aaffkuh_V15312_A	Embrapa	*A*. aff. *kuhlmannii*	A	Perennial
Acfhel_V14685_Grif7526_A	Embrapa	*A*. cf. *helodes*	A	Perennial
helo_V15289_A	Embrapa	*A*. cf. *helodes*	A	Perennial
Acfkuh_V15287_A	Embrapa	*A*. cf. *kuhlmannii*	A	Perennial
Affbat_Wi1300_Grif14260_K	TAMU	*A*. aff. *batizocoi*	K	Annual
Asp1_Ma1470_A	IBONE	*A*. sp. 1	A	Perennial
Asp1_Ma1875_A	IBONE	*A*. sp. 1	A	Perennial
Asp1_Ma1918_A	IBONE	*A*. sp. 1	A	Perennial
Asp2_Ma1904_A	IBONE	*A*. sp. 2	A	Perennial
Asp3_Ma1976_A	IBONE	*A*. sp. 3	A	Perennial
Asp3_Se5020_A	IBONE	*A*. sp. 3	A	Perennial
Asp3_Se5021_A	IBONE	*A*. sp. 3	A	Perennial
Asp4_Se3006_B	IBONE	*A*. sp. 4	B	Annual
Asp4_Se3625_B	Embrapa	*A*. sp. 4	B	Annual
Asp5_Se5011_A	IBONE	*A*. sp. 5	A	Perennial
Asp6_Ma1469_Unk	IBONE	*A*. sp. 6	Unk	Annual
Asp7_Se3740_Unk	IBONE	*A*. sp. 7	Unk	Perennial
Asp9_Ma1979_A	IBONE	*A*. sp. 9	A	Perennial
Asp10_Wi1293_Grif14267_Unk	GRIN‐USDA	*A*. sp. 10	Unk	Unknown
bati_K30079_PI468325_K	GRIN‐USDA	*A*. *batizocoi*	K	Annual
bati_K30080_PI468326_K	NCSU	*A*. *batizocoi*	K	Annual
bati_K30082_PI468328_K	GRIN‐USDA	*A*. *batizocoi*	K	Annual
bati_K30083_PI468329_K	GRIN‐USDA	*A*. *batizocoi*	K	Annual
bati_K9484_PI298639_K	Embrapa	*A*. *batizocoi*	K	Annual
bati_Wi1265_Grif14248_K	GRIN‐USDA	*A*. *batizocoi*	K	Annual
bati_Wi1266_PI686976_K	GRIN‐USDA	*A*. *batizocoi*	K	Annual
bati_Wi1279_Grif14250_K	GRIN‐USDA	*A*. *batizocoi*	K	Annual
bati_Wi1282_PI686977_K	GRIN‐USDA	*A*. *batizocoi*	K	Annual
bati_Wi1284_PI686978_K	GRIN‐USDA	*A*. *batizocoi*	K	Annual
bati_Wi1504_PI666081_K	GRIN‐USDA	*A*. *batizocoi*	K	Annual
bati_Wi1505_PI688944_K	NCSU	*A*. *batizocoi*	K	Annual
bati_Wi1508_PI686979_K	NCSU	*A*. *batizocoi*	K	Annual
bene_K35005_PI475877_F	Embrapa	*A*. *benensis*	F	Annual
bene_K35006_PI475878_F	NCSU	*A*. *benensis*	F	Annual
bene_K35007_PI475879_F	NCSU	*A*. *benensis*	F	Annual
bene_Wi860_PI540870_F	NCSU	*A*. *benensis*	F	Annual
card_G10017_PI262141_A	Embrapa	*A*. *cardenasii*	A	Perennial
card_K36015_PI475994_A	NCSU	*A*. *cardenasii*	A	Perennial
card_K36016_PI475995_A	GRIN‐USDA	*A*. *cardenasii*	A	Perennial
card_K36017_PI475996_A	TAMU	*A*. *cardenasii*	A	Perennial
card_K36018_PI475997_A	TAMU	*A*. *cardenasii*	A	Perennial
card_K36019_PI475998_A	TAMU	*A*. *cardenasii*	A	Perennial
card_K36020_PI475999_A	TAMU	*A*. *cardenasii*	A	Perennial
card_K36021_PI476000_A	TAMU	*A*. *cardenasii*	A	Perennial
card_K36023_PI476002_A	TAMU	*A*. *cardenasii*	A	Perennial
card_K36032_PI476011_A	TAMU	*A*. *cardenasii*	A	Perennial
card_K36032yf_PI476011_A	TAMU	*A*. *cardenasii*	A	Perennial
card_K36033_PI476012_A	GRIN‐USDA	*A*. *cardenasii*	A	Perennial
card_K36034yf_PI476013_A	TAMU	*A*. *cardenasii*	A	Perennial
card_K36035yf_PI261868_A	TAMU	*A*. *cardenasii*	A	Perennial
card_Se3733_A	IBONE	*A*. *cardenasii*	A	Perennial
card_Wi1285_Grif14253_A	NCSU	*A*. *cardenasii*	A	Perennial
card_Wi1286_Grif14254_A	NCSU	*A*. *cardenasii*	A	Perennial
card_Wi1287B_Grif14275_A	GRIN‐USDA	*A*. *cardenasii*	A	Perennial
card_Wi1290_PI681078_A	NCSU	*A*. *cardenasii*	A	Perennial
chiq_K36027_PI476006_A	GRIN‐USDA	*A*. *chiquitana*	A	Perennial
chiq_K36031_PI476010_A	GRIN‐USDA	*A*. *chiquitana*	A	Perennial
corr_G9530_PI262808_A	NCSU	*A*. *correntina*	A	Perennial
corr_G9548_PI262881_A	Embrapa	*A*. *correntina*	A	Perennial
corr_K36000_PI475880_A	NCSU	*A*. *correntina*	A	Perennial
corr_K7830_PI261871_A	TAMU	*A*. *correntina*	A	Perennial
corr_K7897_PI261868_A	NCSU	*A*. *correntina*	A	Perennial
corr_La135_A	Embrapa	*A*. *correntina*	A	Perennial
corr_Se4012_A	IBONE	*A*. *correntina*	A	Perennial
corr_Se4026_A	IBONE	*A*. *correntina*	A	Perennial
corr_Se4027_A	IBONE	*A*. *correntina*	A	Perennial
cruz_K36024_PI476003_K	GRIN‐USDA	*A*. *cruziana*	K	Annual
cruz_K36026_PI476005_K	TAMU	*A*. *cruziana*	K	Annual
cruz_Wi1302_PI688947_K	UGA	*A*. *cruziana*	K	Annual
deco_Sv4533_G	Embrapa	*A*. *decora*	G	Annual
deco_Sv8188_G	Embrapa	*A*. *decora*	G	Annual
deco_V12893_Grif7434_G	TAMU	*A*. *decora*	G	Annual
deco_V12900_PI591342_G	NCSU	*A*. *decora*	G	Annual
deco_V9955_PI666082_G	Embrapa	*A*. *decora*	G	Annual
deco_W674_G	Embrapa	*A*. *decora*	G	Annual
diog_G10602_PI276235_A	Embrapa	*A*. *diogoi*	A	Perennial
diog_K30005_PI468142_A	TAMU	*A*. *diogoi*	A	Perennial
diog_K30106_PI468354_A	IBONE	*A. diogoi*	A	Perennial
diog_Ma1907_A	IBONE	*A. diogoi*	A	Perennial
diog_Vp5000_A	Embrapa	*A. diogoi*	A	Perennial
dura_G10038LL_PI262133_A	GRIN‐USDA	*A. duranensis*	A	Annual
dura_K30061_PI468198_A	GRIN‐USDA	*A. duranensis*	A	Annual
dura_K30064_PI468200_A	TAMU	*A. duranensis*	A	Annual
dura_K30065_PI468201_A	GRIN‐USDA	*A. duranensis*	A	Annual
dura_K30067_PI468202_A	TAMU	*A. duranensis*	A	Annual
dura_K30068_PI468203_A	NCSU	*A. duranensis*	A	Annual
dura_K30069_PI475844_A	GRIN‐USDA	*A. duranensis*	A	Annual
dura_K30070_PI475845_A	GRIN‐USDA	*A. duranensis*	A	Annual
dura_K30071_PI475846_A	NCSU	*A. duranensis*	A	Annual
dura_K30072_PI475847_A	GRIN‐USDA	*A. duranensis*	A	Annual
dura_K30073_PI468319_A	GRIN‐USDA	*A. duranensis*	A	Annual
dura_K30074_PI468320_A	GRIN‐USDA	*A. duranensis*	A	Annual
dura_K30075_PI468321_A	NCSU	*A. duranensis*	A	Annual
dura_K30077_PI468323_A	NCSU	*A. duranensis*	A	Annual
dura_K30078_PI468324_A	GRIN‐USDA	*A. duranensis*	A	Annual
dura_K36002_PI475882_A	GRIN‐USDA	*A. duranensis*	A	Annual
dura_K36003_PI475883_A	GRIN‐USDA	*A. duranensis*	A	Annual
dura_K36005_PI475885_A	GRIN‐USDA	*A. duranensis*	A	Annual
dura_K36036_PI475887_A	GRIN‐USDA	*A. duranensis*	A	Annual
dura_K38900_PI497266_A	GRIN‐USDA	*A. duranensis*	A	Annual
dura_K38903_PI497267_A	GRIN‐USDA	*A. duranensis*	A	Annual
dura_K38904_PI497268_A	GRIN‐USDA	*A. duranensis*	A	Annual
dura_K38906_PI497270_A	GRIN‐USDA	*A. duranensis*	A	Annual
dura_K7988_PI219823_A	Embrapa	*A. duranensis*	A	Annual
dura_Sc21763_PI497262_A	GRIN‐USDA	*A. duranensis*	A	Annual
dura_Sc21764_PI497263_A	GRIN‐USDA	*A. duranensis*	A	Annual
dura_Sc21766_PI497264_A	GRIN‐USDA	*A. duranensis*	A	Annual
dura_Sc21767_PI497265_A	GRIN‐USDA	*A. duranensis*	A	Annual
dura_Se2737_A	Embrapa	*A. duranensis*	A	Annual
dura_Se2845_A	Embrapa	*A. duranensis*	A	Annual
dura_Se2848_A	Embrapa	*A. duranensis*	A	Annual
dura_V14167_A	GRIN‐USDA	*A. duranensis*	A	Annual
dura_Wi1268_PI688950_A	GRIN‐USDA	*A. duranensis*	A	Annual
dura_Wi1270_PI686980_A	GRIN‐USDA	*A. duranensis*	A	Annual
dura_Wi1272_Grif14265_A	GRIN‐USDA	*A. duranensis*	A	Annual
dura_Wi1274_PI688951_A	GRIN‐USDA	*A. duranensis*	A	Annual
dura_Wi1275_PI686981_A	GRIN‐USDA	*A. duranensis*	A	Annual
dura_Wi1506‐E_PI666085_A	GRIN‐USDA	*A. duranensis*	A	Annual
dura_Wi1506‐W_PI688952_A	GRIN‐USDA	*A. duranensis*	A	Annual
dura_Wi1507_PI688953_A	GRIN‐USDA	*A. duranensis*	A	Annual
dura_Wi1510‐B_PI666084_A	GRIN‐USDA	*A. duranensis*	A	Annual
glan_K30091_PI468336_D	TAMU	*A. glandulifera*	D	Annual
glan_K30098_PI468341_D	TAMU	*A. glandulifera*	D	Annual
glan_K30099_PI468342_D	NCSU	*A. glandulifera*	D	Annual
glan_K30100_PI468343_D	TAMU	*A. glandulifera*	D	Annual
glan_V13738_PI599182_D	Embrapa	*A. glandulifera*	D	Annual
glan_V14730_D	Embrapa	*A. glandulifera*	D	Annual
greg_V6390_PI476117_B	Embrapa	*A. gregoryi*	B	Annual
greg_V6389_PI476116_B	GRIN‐USDA	*A. gregoryi*	B	Annual
helo_K30029_PI468144_A‐2	NCSU	*A. helodes*	A	Perennial
helo_K30029_PI468144_A	UGA	*A. helodes*	A	Perennial
helo_K30031_PI468146_A	GRIN‐USDA	*A. helodes*	A	Perennial
helo_K30036yf_PI468147_A	GRIN‐USDA	*A. helodes*	A	Perennial
helo_V10470_A	Embrapa	*A. helodes*	A	Perennial
helo_V12083_A	Embrapa	*A. helodes*	A	Perennial
helo_V14678_A	Embrapa	*A. helodes*	A	Perennial
helo_V6325_A	Embrapa	*A. helodes*	A	Perennial
helo_V6326_PI476043_A	GRIN‐USDA	*A. helodes*	A	Perennial
helo_V6330_PI476044_A	GRIN‐USDA	*A. helodes*	A	Perennial
helo_V6331_PI476045_A	TAMU	*A. helodes*	A	Perennial
herz_Se3334_A	IBONE	*A. herzogii*	A	Perennial
hoeh_K30006_PI468150_Unk	GRIN‐USDA	*A. hoehnei*	B	Annual
hoeh_V13985_Unk	Embrapa	*A. hoehnei*	B	Annual
hoeh_V14547_Unk	Embrapa	*A. hoehnei*	B	Annual
hoeh_V9094_PI688954_Unk	Embrapa	*A. hoehnei*	B	Annual
hoeh_V9140_PI666086_Unk	Embrapa	*A. hoehnei*	B	Annual
hoeh_V9146_PI681081_Unk	NCSU	*A. hoehnei*	B	Annual
infl_Se3292_B	Embrapa	*A. inflata*	B	Annual
infl_Se3637_B	IBONE	*A. inflata*	B	Annual
ipae_K30076_PI468322_B	TAMU	*A. ipaënsis*	B	Annual
kemp_K35001_PI475873_A	TAMU	*A. kempff‐mercadoi*	A	Perennial
kemp_Se3170_A	IBONE	*A. kempff‐mercadoi*	A	Perennial
kemp_V13250_A	Embrapa	*A. kempff‐mercadoi*	A	Perennial
kemp_V14673_A	Embrapa	*A. kempff‐mercadoi*	A	Perennial
kemp_V14886_A	Embrapa	*A. kempff‐mercadoi*	A	Perennial
kemp_V15135_A	Embrapa	*A. kempff‐mercadoi*	A	Perennial
krap_Wi1287A_Grif14274_K	GRIN‐USDA	*A. krapovickasii*	K	Annual
krap_Wi1288_Grif14258_K	GRIN‐USDA	*A. krapovickasii*	K	Annual
krap_Wi1288‐2_Grif14258_K	GRIN‐USDA	*A. krapovickasii*	K	Annual
krap_Wi1291_K	TAMU	*A. krapovickasii*	K	Annual
kuhl_K30008_PI468152_A	NCSU	*A. kuhlmannii*	A	Perennial
kuhl_K30017_PI468159_A	TAMU	*A. kuhlmannii*	A	Perennial
kuhl_K30034yf_PI468167_A	TAMU	*A. kuhlmannii*	A	Perennial
kuhl_V10506_Grif7734_A	Embrapa	*A. kuhlmannii*	A	Perennial
kuhl_V13530_A	Embrapa	*A. kuhlmannii*	A	Perennial
kuhl_V13721_A	Embrapa	*A. kuhlmannii*	A	Perennial
kuhl_V13736_A	TAMU	*A. kuhlmannii*	A	Perennial
kuhl_V14691_A	Embrapa	*A. kuhlmannii*	A	Perennial
kuhl_V14705_A	Embrapa	*A. kuhlmannii*	A	Perennial
kuhl_V15296_A	Embrapa	*A. kuhlmannii*	A	Perennial
kuhl_V15299_A	Embrapa	*A. kuhlmannii*	A	Perennial
kuhl_V6351_PI476108_A	NCSU	*A. kuhlmannii*	A	Perennial
kuhl_V6352_A	Embrapa	*A. kuhlmannii*	A	Perennial
kuhl_V6355_PI476110_A	TAMU	*A. kuhlmannii*	A	Perennial
kuhl_V6380_PI476115_A	Embrapa	*A. kuhlmannii*	A	Perennial
kuhl_V6404_PI476119_A	Embrapa	*A. kuhlmannii*	A	Perennial
kuhl_V6413_PI476126_A	Embrapa	*A. kuhlmannii*	A	Perennial
kuhl_V7639_PI681082_A	GRIN‐USDA	*A. kuhlmannii*	A	Perennial
kuhl_V8753_Grif7648_A	GRIN‐USDA	*A. kuhlmannii*	A	Perennial
kuhl_V8887_A	Embrapa	*A. kuhlmannii*	A	Perennial
kuhl_V8916_PI666094_A	GRIN‐USDA	*A. kuhlmannii*	A	Perennial
kuhl_V8916a_PI666094_A	Embrapa	*A. kuhlmannii*	A	Perennial
kuhl_V8979_PI604827_A	Embrapa	*A. kuhlmannii*	A	Perennial
kuhl_V9214_PI666089_A	Embrapa	*A. kuhlmannii*	A	Perennial
kuhl_V9230_PI666090_A	Embrapa	*A. kuhlmannii*	A	Perennial
kuhl_V9230_PI666090_A‐2	NCSU	*A. kuhlmannii*	A	Perennial
kuhl_V9235_Grif7692_A	Embrapa	*A. kuhlmannii*	A	Perennial
kuhl_V9243_PI666091_A	Embrapa	*A. kuhlmannii*	A	Perennial
kuhl_V9375_Grif7698_A	Embrapa	*A. kuhlmannii*	A	Perennial
kuhl_V9394_A	Embrapa	*A. kuhlmannii*	A	Perennial
kuhl_V9470_PI686982_A	Embrapa	*A. kuhlmannii*	A	Perennial
kuhl_V9479_PI688955_A	Embrapa	*A. kuhlmannii*	A	Perennial
kuhl_V9912_Grif7712_A	Embrapa	*A. kuhlmannii*	A	Perennial
line_V9401_Grif7700_A	Embrapa	*A. linearifolia*	A	Perennial
magn_K30092_PI468337_B	TAMU	*A. magna*	B	Annual
magn_K30093_PI468338_B	TAMU	*A. magna*	B	Annual
magn_K30097_PI468340_B	Embrapa	*A. magna*	B	Annual
magn_K30097yf_PI468340_B	TAMU	*A. magna*	B	Annual
magn_Se3777_B	IBONE	*A. magna*	B	Annual
magn_V13751_B	Embrapa	*A. magna*	B	Annual
magn_V13752_PI599183_B	TAMU	*A. magna*	B	Annual
magn_V13760_PI599184_B	TAMU	*A. magna*	B	Annual
magn_V13761_B	Embrapa	*A. magna*	B	Annual
magn_V6396_PI476118_B	TAMU	*A. magna*	B	Annual
micr_Cb962_PI666096_A	GRIN‐USDA	*A. microsperma*	A	Perennial
micr_V14031_A	Embrapa	*A. microsperma*	A	Perennial
micr_V14042_A	Embrapa	*A. microsperma*	A	Perennial
palu_V13023_PI666093_G	Embrapa	*A. palustris*	G	Annual
palu_V6611_G	TAMU	*A. palustris*	G	Annual
prae_V13777_G	TAMU	*A. praecox*	G	Annual
prae_V6416_PI476128_G	Embrapa	*A. praecox*	G	Annual
schi_V9923_Grif7716_A	Embrapa	*A. schininii*	A	Annual
simp_K36010_A	TAMU	*A. simpsonii*	A	Perennial
simp_V13710_PI674413_A	Embrapa	*A. simpsonii*	A	Perennial
simp_V13728_A	Embrapa	*A. simpsonii*	A	Perennial
simp_V13732_PI688958_A	GRIN‐USDA	*A. simpsonii*	A	Perennial
step_HLK408_PI338279_A	Embrapa	*A. stenosperma*	A	Short‐lived perennial
step_HLK410_PI338280_A	TAMU	*A. stenosperma*	A	Short‐lived perennial
step_Sv3712_A	Embrapa	*A. stenosperma*	A	Short‐lived perennial
step_V10229_A	Embrapa	*A. stenosperma*	A	Short‐lived perennial
step_V10309_PI666100_A	Embrapa	*A. stenosperma*	A	Short‐lived perennial
step_V12488_A	Embrapa	*A. stenosperma*	A	Short‐lived perennial
step_V12575_PI591359_A	Embrapa	*A. stenosperma*	A	Short‐lived perennial
step_V12646_Grif7750_A	GRIN‐USDA	*A. stenosperma*	A	Short‐lived perennial
step_V13260_PI666098_A	GRIN‐USDA	*A. stenosperma*	A	Short‐lived perennial
step_V13262_PI591351_A	GRIN‐USDA	*A. stenosperma*	A	Short‐lived perennial
step_V13267_PI591352_A	GRIN‐USDA	*A. stenosperma*	A	Short‐lived perennial
step_V13672_PI599179_A	GRIN‐USDA	*A. stenosperma*	A	Short‐lived perennial
step_V13693_PI599180_A	Embrapa	*A. stenosperma*	A	Short‐lived perennial
step_V13796_PI599185_A	Embrapa	*A. stenosperma*	A	Short‐lived perennial
step_V13824_PI599186_A	GRIN‐USDA	*A. stenosperma*	A	Short‐lived perennial
step_V13828_PI599187_A	NCSU	*A. stenosperma*	A	Short‐lived perennial
step_V13840_A	TAMU	*A. stenosperma*	A	Short‐lived perennial
step_V13844_PI599189_A	TAMU	*A. stenosperma*	A	Short‐lived perennial
step_V14092_A	Embrapa	*A. stenosperma*	A	Short‐lived perennial
step_V14453_A	Embrapa	*A. stenosperma*	A	Short‐lived perennial
step_V14454_A	Embrapa	*A. stenosperma*	A	Short‐lived perennial
step_V14773_A	Embrapa	*A. stenosperma*	A	Short‐lived perennial
step_V15076_A	Embrapa	*A. stenosperma*	A	Short‐lived perennial
step_V7377_PI497578_A	GRIN‐USDA	*A. stenosperma*	A	Short‐lived perennial
step_V7379_PI497579_A	Embrapa	*A. stenosperma*	A	Short‐lived perennial
step_V7382_PI497580_A	GRIN‐USDA	*A. stenosperma*	A	Short‐lived perennial
step_V7384_PI497581_A	GRIN‐USDA	*A. stenosperma*	A	Short‐lived perennial
step_V7762_PI681084_A	Embrapa	*A. stenosperma*	A	Short‐lived perennial
step_V9010_Grif7677_A	GRIN‐USDA	*A. stenosperma*	A	Short‐lived perennial
step_V9012_Grif7678_A	GRIN‐USDA	*A. stenosperma*	A	Short‐lived perennial
step_V9017_PI666099_A	GRIN‐USDA	*A. stenosperma*	A	Short‐lived perennial
step_Ve13256_PI666097_A	GRIN‐USDA	*A. stenosperma*	A	Short‐lived perennial
step_Ve13258_PI591350_A	Embrapa	*A. stenosperma*	A	Short‐lived perennial
step_W422_A	Embrapa	*A. stenosperma*	A	Short‐lived perennial
trin_Wi1117_PI666101_F	IBONE	*A. trinitensis*	F	Annual
vali_K30011_PI468154_B	GRIN‐USDA	*A. valida*	B	Annual
vali_K30147_PI468184_B	TAMU	*A. valida*	B	Annual
vali_V13514_B	Embrapa	*A. valida*	B	Annual
vali_V9153_PI666102_B	GRIN‐USDA	*A. valida*	B	Annual
vali_V9157_PI666103_B	NCSU	*A. valida*	B	Annual
vals_V13515_Unk	Embrapa	*A. vallsii*	ukn	Annual
vals_V7635_Unk	Embrapa	*A. vallsii*	ukn	Annual
villo_50949_A	TAMU	*A. villosa*	A	Perennial
villo_Mt1_PI666104_A	NCSU	*A. villosa*	A	Perennial
villo_Mt2_PI666105_A	GRIN‐USDA	*A. villosa*	A	Perennial
villo_Mt3_PI666106_A	GRIN‐USDA	*A. villosa*	A	Perennial
villo_Mt4_PI666107_A	GRIN‐USDA	*A. villosa*	A	Perennial
villo_Mt5_PI666108_A	GRIN‐USDA	*A. villosa*	A	Perennial
villo_V12812_A	Embrapa	*A. villosa*	A	Perennial
villo_V14316_A	Embrapa	*A. villosa*	A	Perennial
will_Wi1118_PI688988_B	Embrapa	*A. williamsii*	B	Annual

**Table 2:** List of *Arachis* genome sequences used for *in silico* SNP extraction. SNPs were extracted separately from the component subgenomes of the *Arachis hypogaea* sequences. The diploid wild species genome sequences were used to perform a pairwise alignment of *in silico* extracted SNPs with Axiom_Arachis v02 determined SNPs to test the reproducibility of the different dataset types

Manuscript ID	Original ID	Species and subspecies	Botanical variety	Genbank	Link to genome sequence
hypo.Tifrunner	arahy.Tifrunner.gnm2	*Arachis hypogaea* subsp. *hypogaea*	*hypogaea*	GCA_003086295.3	https://data.legumeinfo.org/Arachis/hypogaea/genomes/Tifrunner.gnm2.J5K5/
hypo.TennRed	arahy.TennRed.gnm1	*Arachis hypogaea* subsp. *fastigiata*	*fastigiata*	GCA_022829005.1	https://www.ncbi.nlm.nih.gov/bioproject/PRJNA801227
hypo.Shitouqi	Peanut Shitouq	*Arachis hypogaea* subsp. *fastigiata*	*vulgaris*	GCA_003713155.1	https://data.legumeinfo.org/Arachis/hypogaea/genomes/Shitouqi.gnm1.L4VP/
hypo.Fuhuasheng	arahy.Fuhuasheng.gnm1.XX5Y	*Arachis hypogaea* subsp. *fastigiata*	*vulgaris*	GCA_004170445.1	https://data.legumeinfo.org/Arachis/hypogaea/genomes/Fuhuasheng.gnm1.XX5Y/
hypo.Haihua	Haihua1	*Arachis hypogaea* subsp. *fastigiata*	*vulgaris*	GCA_016103905.1	https://www.ncbi.nlm.nih.gov/bioproject/PRJNA509953/
ipae.K30076.gnm2	araip.K30076.gnm2.1GWY	*Arachis ipaënsis*		GCA_000816755.2	https://data.legumeinfo.org/Arachis/ipaensis/genomes/K30076.gnm2.1GWY/
step.V10309.gnm1	arast.V10309.gnm1.PFL2	*Arachis stenosperma*		GCA_014773155.1	https://data.legumeinfo.org/Arachis/stenosperma/genomes/V10309.gnm1.PFL2/
dura.V14167.gnm2	aradu.V14167.gnm2.J7QH	*Arachis duranensis*		GCA_000817695.3	https://data.legumeinfo.org/Arachis/duranensis/genomes/V14167.gnm2.J7QH/
dura.K30060.gnm1	aradu.K30060.gnm1.W9B1	*Arachis duranensis*		GCA_018207795.1	https://www.ncbi.nlm.nih.gov/datasets/genome/GCA_018207795.1/
dura.K30065.gnm1	aradu.K30065.gnm1.M24N	*Arachis duranensis*		GCA_014805325.1	https://www.ncbi.nlm.nih.gov/datasets/genome/GCA_014805325.1/

To test the quality of the *in silico* SNP detection and assess reproducibility, a pairwise alignment was conducted using the MUSCLE5.1 multi‐alignment tool with the Super5 algorithm (Edgar, 2004). This alignment used concatenated SNP pseudosequences extracted from five high‐quality genome sequences of wild diploid accessions—*A. duranensis* K 30060, *A. duranensis* K 30065, *A. duranensis* V 14167, *A. ipaënsis* K 30076, and *A. stenosperma* V 10309 (Table 2)—and their corresponding concatenated Affymetrix‐derived SNP pseudosequences for those same reference accession samples from Leal‐Bertioli et al. (2024).

### *Arachis duranensis* K 30065 genome assembly and scaffolding

High molecular weight DNA was extracted from frozen tissue into CTAB buffer (that included proteinase K, PVP‐40 and beta‐mercaptoethanol) for 1 h at 50°C as previously described (Vaughn et al., 2021). Libraries were prepared using the PacBio SMRTbell Template Prep Kit 1.0, PacBio SMRTbell Damage Repair Kit, and prepared for sequencing using the PacBio DNA/Polymerase Binding Kit P6 V2. Sequencing was performed on the PacBio SequelI. All protocols used were PacBio recommended protocols. Scaffolds were placed into pseudomolecules based on synteny with three other A genome *Arachis* assemblies: *A. duranensis* K 30065 (GCA_014805325.1), *A. duranensis* V 14167 (GCA_026016865.1), and *A. hypogaea* chromosomes 1‐10 (GCA_003086295.3). Genome comparisons and synteny were assessed using Mummer4 (Marçais et al., 2018). Five apparent scaffold mis‐joins were identified and corrected (in scaffolds tig00061192, tig00723559, tig00061205, tig00061202, tig00061237). The pseudomolecules are comprised of 73 scaffolds, with 64 within‐chromosome gaps (each represented by 100 Ns). The total assembly size is 1099 Mb, of which 1086 Mb is in 10 pseudomolecules, and 13 Mb remains in 112 unanchored scaffolds.

### Variant analysis

*Arachis hypogaea* cv. Tifrunner (https://data.legumeinfo.org/Arachis/hypogaea/genomes/Tifrunner.gnm2.J5K5/), *A. duranensis* K 30065 (Río Seco, https://www.ncbi.nlm.nih.gov/datasets/genome/GCA_014805325.1/), and *A. duranensis* V 14167 (https://data.legumeinfo.org/Arachis/duranensis/genomes/V14167.gnm2.J7QH/) were aligned together using the minigraph‐cactus pipeline (Hickey et al., 2024). Tifrunner was used as the initial reference genome. We used minigraph‐cactus to generate a VCF file (‐‐vcf clip) from the resultant alignment containing graph‐based variant calls in the Tifrunner coordinate system. Using bcftools 1.19 (Danecek et al., 2021), V 14167‐specific variants were defined as variants, where V14167 was explicitly called as an alternate allele, and K 30065 was also explicitly called as the reference allele. K 30065‐specific alleles were defined in a similar manner. These lineage‐specific variants in 2 MB windows (also, in 100 Kb windows for a fine‐grain resolution, Data S1) were counted and plotted across the genome using bedtools v2.31.0 (Quinlan & Hall, 2010) and R version 4.4.1. The variant count analysis was also performed only for the Tifrunner genic regions (https://data.legumeinfo.org/Arachis/hypogaea/annotations/Tifrunner.gnm2.ann1.4K0L/).

### Data processing for genetic relationships analysis

**Figure 6:**
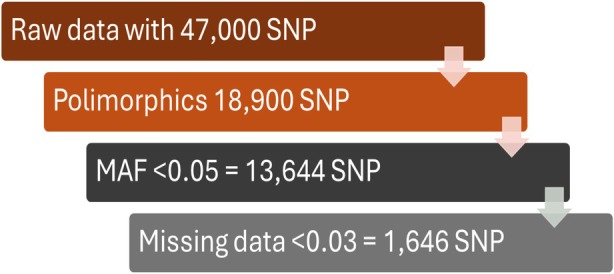
Three‐step filtering process from raw data, to obtain 13 644 SNP markers used to merge the reference panel wild *Arachis* species with the six *Arachis hypogaea* varieties, and finally 1646 SNP for the genetic relationships among the 286 accessions assessed in this work.

The 13 644 curated SNP database from the reference wild species panel was combined with the two data sets obtained from the component tetraploid *Arachis* A and B subgenome markers. The SNP set for each sample was then aligned using the MUSCLE 5.1 multi‐alignment tool (Edgar, 2004). Three steps of filtering were applied after beginning with raw SNP data, as Figure 6 illustrates. Initially, only polymorphic markers remained after the raw data was filtered out. Second, 13 644 curated SNPs were obtained by applying TASSEL 5.0 (Bradbury et al., 2007) to minor allele frequency (MAF) data filtering (<0.05). Finally, using a custom UNIX filter (See Appendix 1) the data was subjected to a final step of filtering for a maximum of 3% of missing data for each sample from which 1646 SNPs were obtained to perform phylogenetic and PCA.

### PCA and genetic tree

Phylogenetic and PCA were performed to estimate the overall relationships among accessions of the wild *Arachis* germplasm, as investigated in Leal‐Bertioli et al. (2024), and the five *A. hypogaea* accessions. PCA scores were calculated and plotted with the biplot() function, using the vegan and PCAtools R packages (Blighe et al., 2019; R Core Team, 2014; Oksanen et al., 2013), based on 281 individuals (272 wild diploids, 5 cultivated A genome SNP set, and 5 cultivated B genome SNP set) and 1682 SNPs. The number of principal components (PCs) explaining >80% of the variation was determined by two methods: (i) the Scree plot (Cattell, 1966) and (ii) the cumulative variance approach (which(cumsum(pcaobject$variance) >80) in R), based on eigenvalues of the Euclidean distance matrix. Pairwise similarity among accessions was additionally quantified using identity‐by‐state (IBS) counts, including both homozygous and heterozygous genotype calls, following Singh et al. (2019) (Data S2). This similarity matrix was used only to summarize genetic relatedness among samples. For phylogeny, SNP alignments were generated with MUSCLE v5.1 (Edgar, 2004) and analyzed with FastTree v2.1.11 (Price et al., 2010), applying the CAT approximation to account for rate heterogeneity among sites. Node support was estimated with 1000 resamplings (Shimodaira & Hasegawa, 1999), and the consensus tree was visualized in FigTree v1.4.4 (Rambaut, 2018). The multialignment used for this analysis is provided as Data S3.

To address the possibility of reticulation due to incomplete lineage sorting or introgression, we also performed a NeighborNet analysis in SplitsTree v6.0.0 (Huson & Bryant, 2024). The resulting split network (Figure S2) was consistent with the maximum likelihood tree, supporting the robustness of the inferred relationships while confirming that a tree‐like representation remains an adequate summary of the data.

## CONFLICT OF INTEREST

The authors have not declared a conflict of interest.

## Supporting information


**Data S1.** Lineage‐specific variant counts of *Arachis duranensis* K30065 (Río Seco, https://www.ncbi.nlm.nih.gov/datasets/genome/GCA_014805325.1/), and *A. duranensis* V 14167 (https://data.legumeinfo.org/Arachis/duranensis/genomes/V14167.gnm2.J7QH/) at Tifrunner genic regions (Peanutbase: arahy.Tifrunner.gnm2.ann1.4K0L.gene_models_main.bed.gz) in 2 MB windows and 100 Kb windows for a fine‐grain resolution. The variants were counted and used to produce Figures 3 and 4 and Figure S1.

**Data S2.** Identity matrix of 281 accessions of 47 diploid species and 5 cultivated. 281 individuals (272 accessions of 47 wild diploids species, 5 cultivated A genome SNP set, and 5 cultivated B genome SNP set).

**Data S3.** Multialignment of SNP sequences used for phylogenetic analysis. SNP alignments of 281 *Arachis* accessions were generated with MUSCLE v5.1 (Edgar, 2004). These alignments served as input for maximum #likelihood phylogenetic inference in FastTree v2.1.11 (Price et al., 2010; Figure 4).

**Data S4.** Dataset of 281 accessions from the *Arachis* section used in this work. Information includes Sample ID as shown in this work, species class either wild or cultivated, SNP name as in 47K SNP Axiom_Arachis v02 genotyping platform (Korani et al., 2019). SNP are depicted in IUPAC code.

**Figure S1.** Distribution of ‘N’ (unknown nucleotide) stretches in the genome assembly of *Arachis duranensis* V 14167, K 30065 and K 30060. *x*‐axis represents chromosome position and *y*‐axis represents length of ‘N’ stretches at each chromosome position.

**Figure S2.** Split network of *Arachis* accessions inferred with NeighborNet.

Appendix 1.

## Data Availability

The genome assembly of *Arachis duranensis* K30065 is available at GenBank under accession GCA_014805325.1. Variant density counts of *A. duranensis* K30065 and *A. duranensis* V14167 relative to the *A. hypogaea* reference genome ‘Tifrunner’ are provided in Data S1. The identity‐by‐state (IBS) matrix is available in Data S2, and the pseudosequences used for phylogenetic analysis are provided in Data S3. SNP flanking sequences and base variants for the *A. hypogaea* A and B subgenomes and the wild species accessions are available in Data S4.
