# Supplementary material for: A single hybrid origin of cultivated peanut
Source: Plant J. 2025 Dec 24;124(6):e70619. doi: 10.1111/tpj.70619 (PMC12737836; doi:10.1111/tpj.70619)
Supplement: Supplementary file 5 — Figure S1. Distribution of ‘N’ (unknown nucleotide) stretches in the genome assembly of Arachis duranensis V 14167, K 30065 and K 30060. x‐axis represents chromosome position and y‐axis represents length of ‘N’ stretches at each chromosome position. [file TPJ-124-0-s001.pdf]

Figure S 1: Distribution of "N" (unknown nucleotide) stretches in the genome assembly of V14167, K30065 and K30060. X-axis represents chromosome position and y-axis represents length of "N" stretches at each chromosome position.

V14167

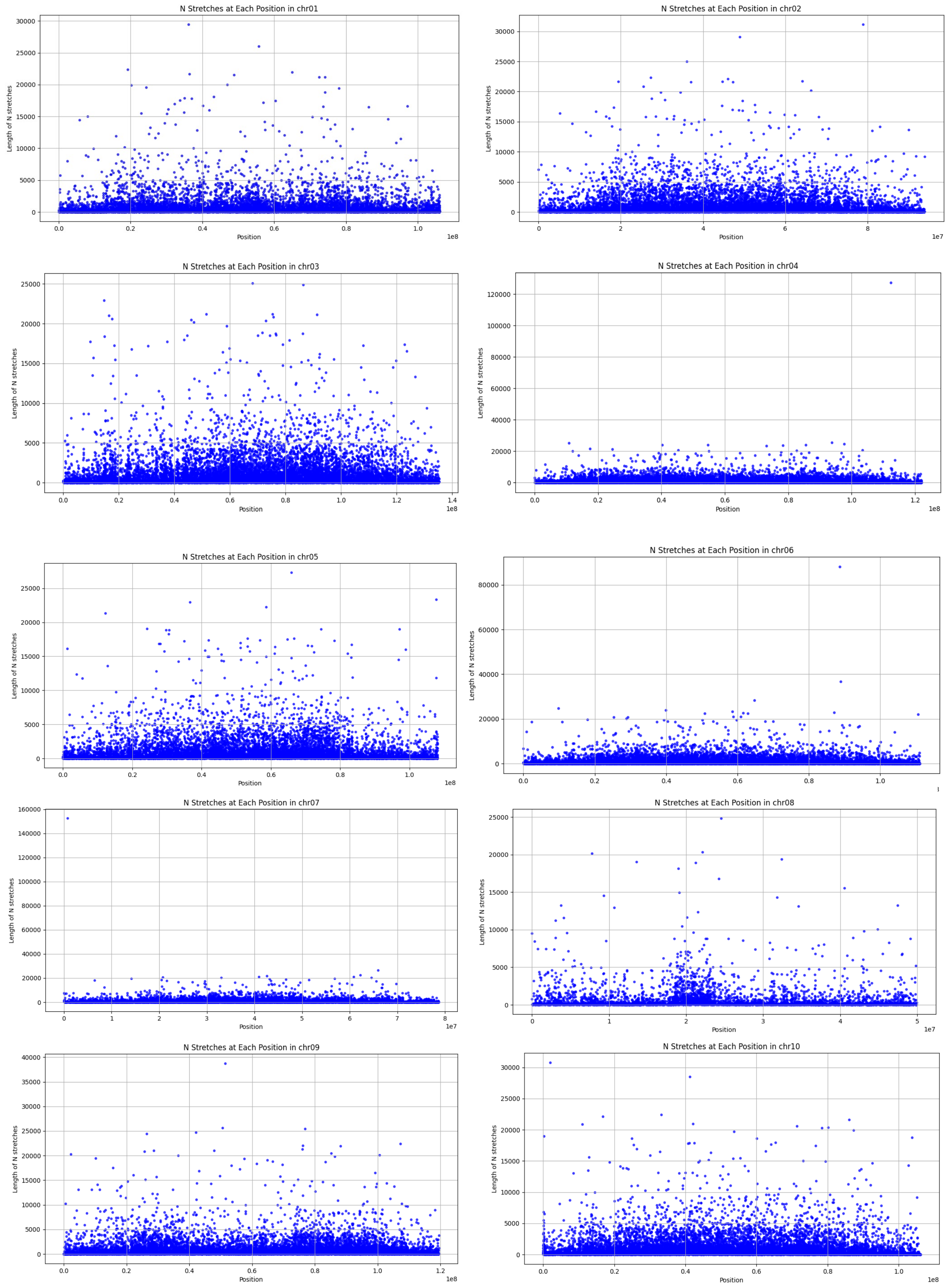

K30065

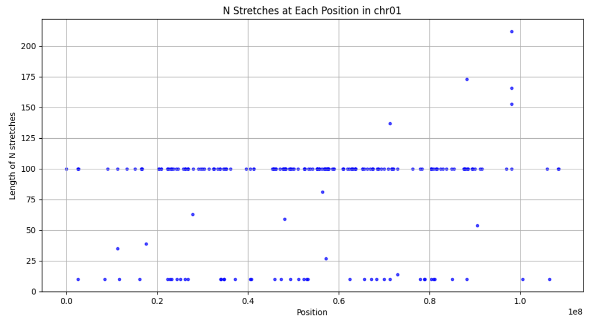
N Stretches at Each Position in chr01
Length of N stretches
Position
1e8

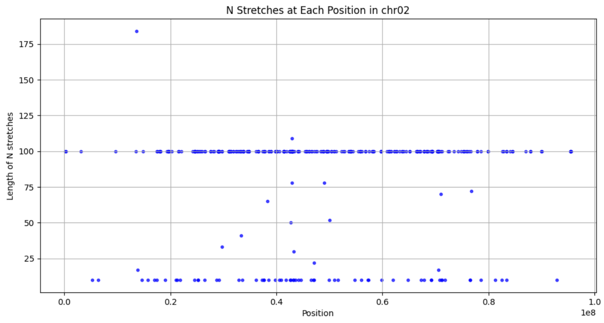
N Stretches at Each Position in chr02
Length of N stretches
Position
1e8

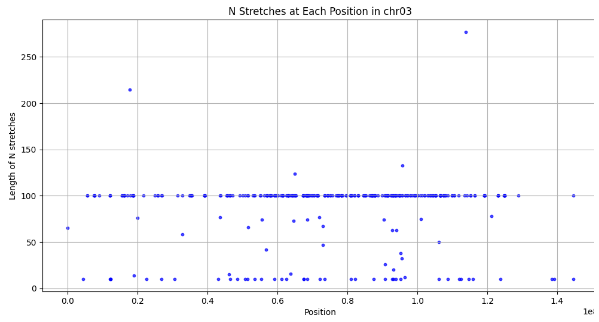
N Stretches at Each Position in chr03
Length of N stretches
Position

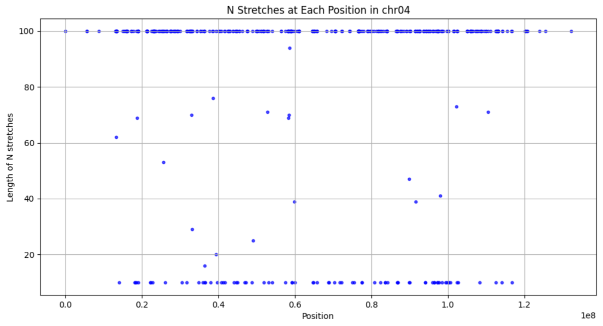
N Stretches at Each Position in chr04
Length of N stretches
Position
1e8

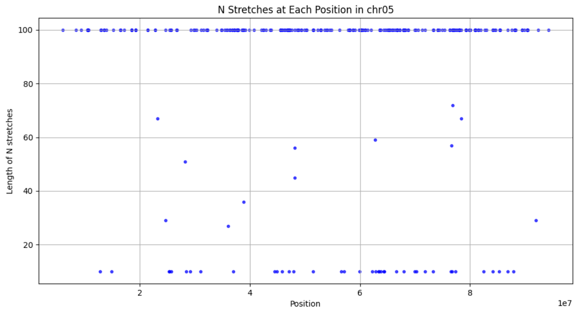
N Stretches at Each Position in chr05
Length of N stretches
Position
1e7

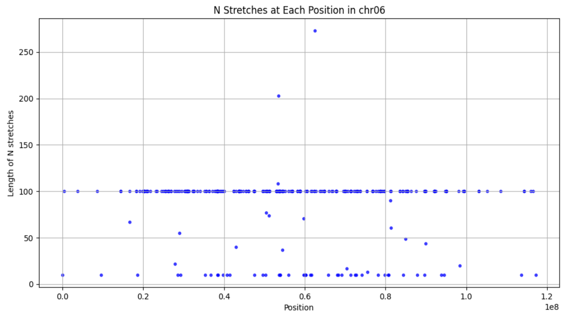
N Stretches at Each Position in chr06
Length of N stretches
Position
1e8

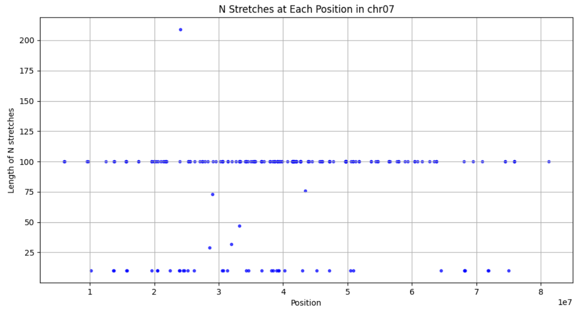
N Stretches at Each Position in chr07
Length of N stretches
Position
1e7

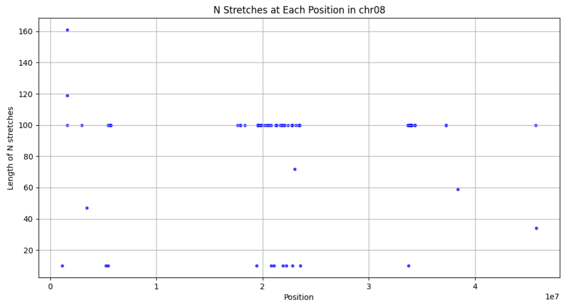
N Stretches at Each Position in chr08
Length of N stretches
Position
1e7

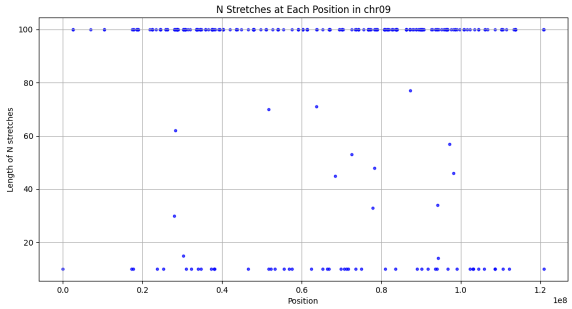
N Stretches at Each Position in chr09
Length of N stretches
Position
1e8

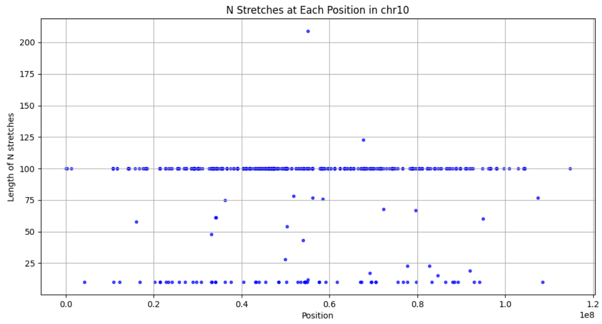
N Stretches at Each Position in chr10
Length of N stretches
Position
1e8

K30060

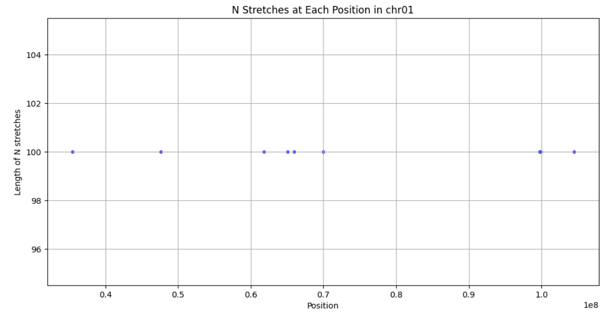
N Stretches at Each Position in chr01
Length of N stretches
Position
1e8
96
98
100
102
104
0.4
0.5
0.6
0.7
0.8
0.9
1.0

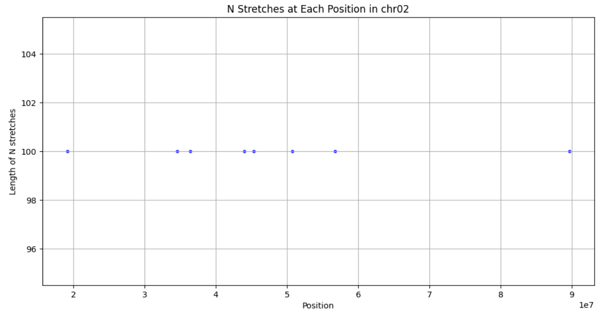
N Stretches at Each Position in chr02
Length of N stretches
Position
1e7
96
98
100
102
104
2
3
4
5
6
7
8
9

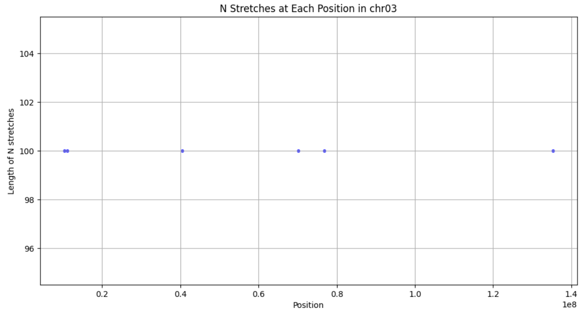
N Stretches at Each Position in chr03
Length of N stretches
Position
1e8
96
98
100
102
104
0.2
0.4
0.6
0.8
1.0
1.2
1.4

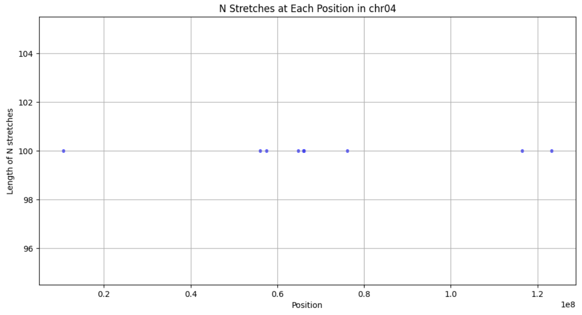
N Stretches at Each Position in chr04
Length of N stretches
Position
1e8
96
98
100
102
104
0.2
0.4
0.6
0.8
1.0
1.2

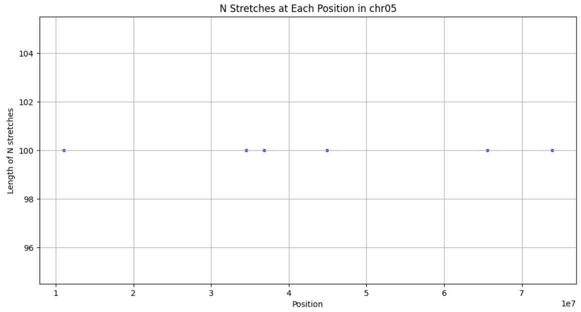
N Stretches at Each Position in chr05
Length of N stretches
Position
1e7
96
98
100
102
104
1
2
3
4
5
6
7

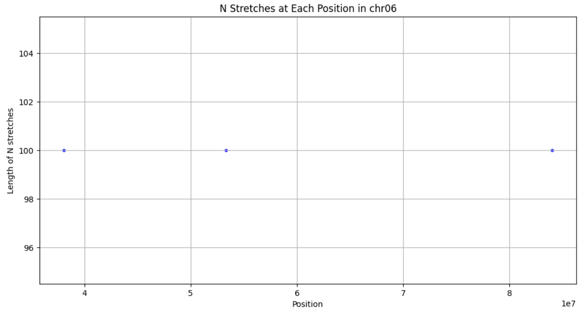
N Stretches at Each Position in chr06
Length of N stretches
Position
1e7
96
98
100
102
104
4
5
6
7
8

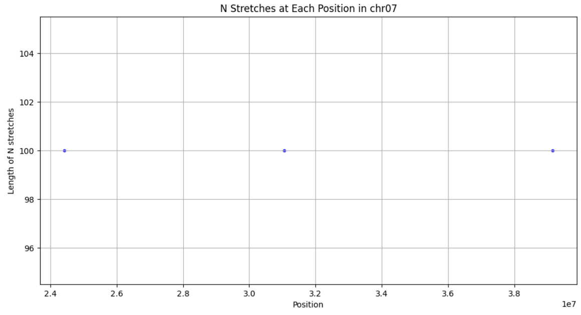
N Stretches at Each Position in chr07
Length of N stretches
Position
1e7
96
98
100
102
104
2.4
2.6
2.8
3.0
3.2
3.4
3.6
3.8

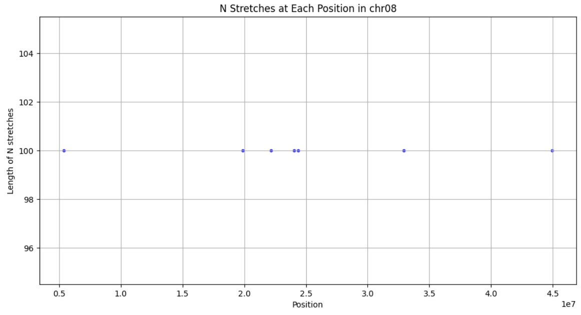
N Stretches at Each Position in chr08
Length of N stretches
Position
1e7
96
98
100
102
104
0.5
1.0
1.5
2.0
2.5
3.0
3.5
4.0
4.5

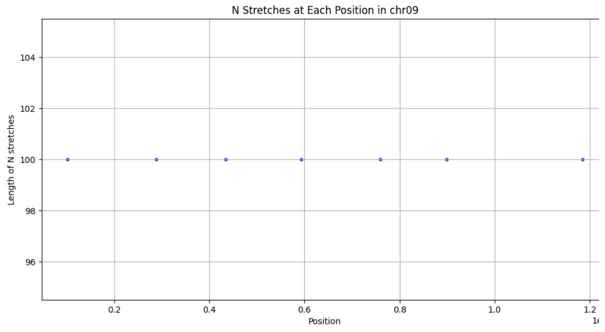
N Stretches at Each Position in chr09
Length of N stretches
Position
96
98
100
102
104
0.2
0.4
0.6
0.8
1.0
1.2

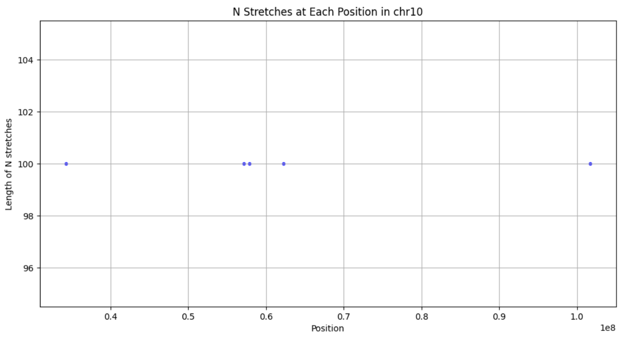
N Stretches at Each Position in chr10
Length of N stretches
Position
1e8
96
98
100
102
104
0.4
0.5
0.6
0.7
0.8
0.9
1.0
