# Supplementary material for: A single hybrid origin of cultivated peanut
Source: Plant J. 2025 Dec 24;124(6):e70619. doi: 10.1111/tpj.70619 (PMC12737836; doi:10.1111/tpj.70619)

Figure S 2. Split network of *Arachis* accessions inferred with NeighborNet. A split network was reconstructed in SplitsTree v6.0.0 (Huson and Bryant, 2024) using the P-distance method (Hamming, 1950) from the SNP dataset comprising 281 accessions (276 wild diploids and 5 cultivated accessions). Visualization was performed with the “Show Splits” function. The network structure was consistent with the maximum likelihood phylogeny, supporting the robustness of the tree-like representation while accounting for potential reticulation.

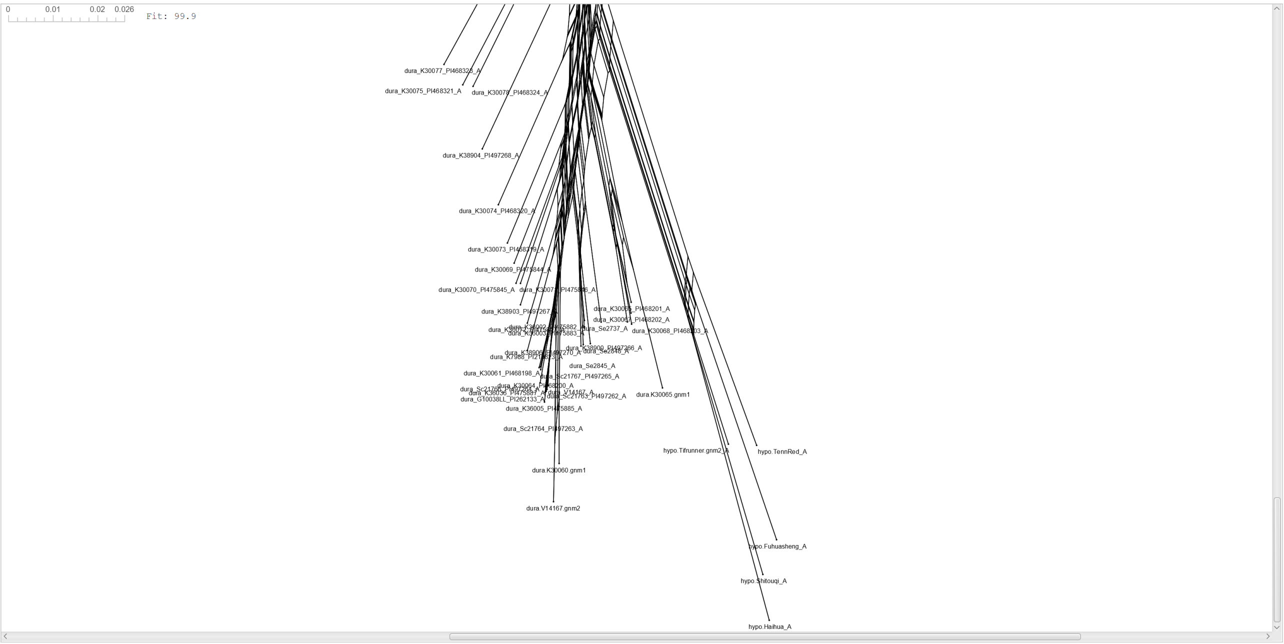

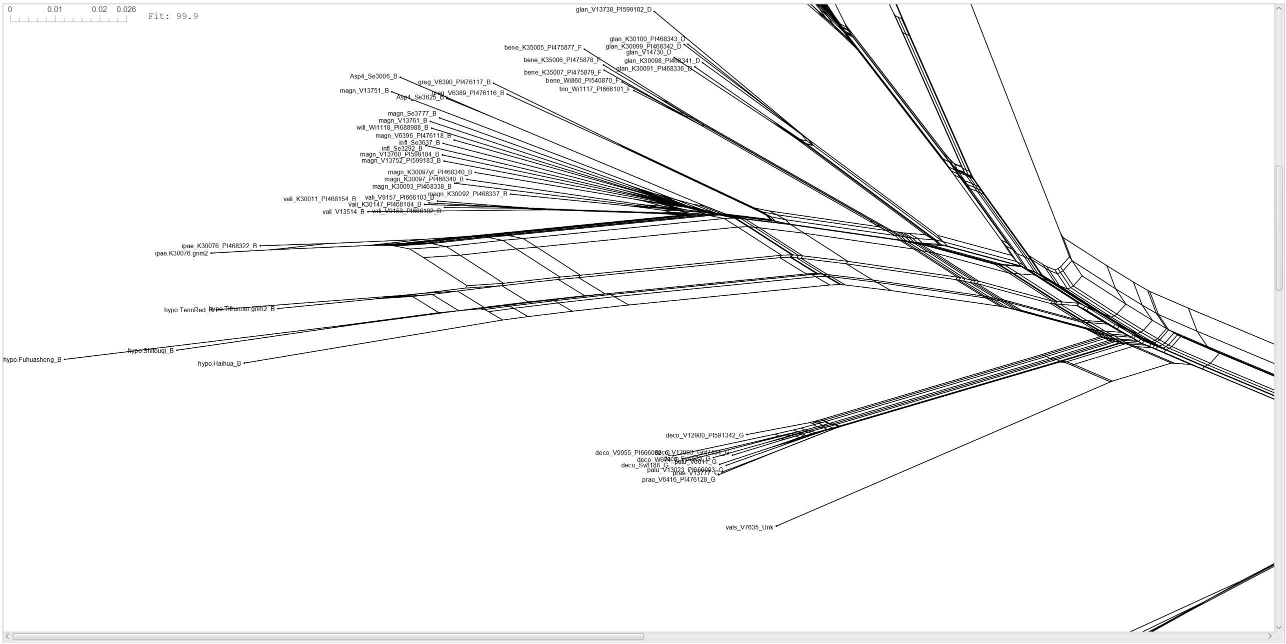

Supplement: Supplementary file 6 — Figure S2. Split network of Arachis accessions inferred with NeighborNet. [file TPJ-124-0-s007.pdf]
